# 639 Trends and Outcomes of Adult Burn Patients with Cannabis Use

**DOI:** 10.1093/jbcr/iraf019.268

**Published:** 2025-04-01

**Authors:** Sarah Wang, Sunnie Wong, Eloise Stanton, Artur Manasyan, Amara Emeh, Elizabeth Boudiab, Deborah Choe, Maxwell Johnson, Haig Yenikomshian, Justin Gillenwater

**Affiliations:** University of California Keck School of Medicine; Los Angeles General Medical Center; University of Southern California; University of California Keck School of Medicine; University of California Keck School of Medicine; Los Angeles General Medical Center Burn Unit; University of California Keck School of Medicine; University of California Keck School of Medicine; University of California Keck School of Medicine; Los Angeles General Medical Center

## Abstract

**Introduction:**

Cannabis use in the U.S. has doubled from 7.6% in 2013 to 15% in 2022, with rates as high as 27% among burn patients in 2011. Despite this trend, the impact of cannabis on burn patients remains underexplored. As cannabis legalization expands, understanding its effects on clinical outcomes is crucial. This study aims to characterize the demographics, hospital course, and outcomes of burn patients who tested positive for cannabis on admission.

**Methods:**

A retrospective study of adult burn patients at a level one trauma center (2015-2024) compared cannabis users, identified via admission urine toxicology, to those negative for illicit drugs. Patients younger than 18 years old and those who had a positive urine drug screen but were cannabis negative were excluded. The primary predictor was cannabis use, with outcomes including hospital length of stay (LOS), morbidities, mortality, discharge status, and readmissions. Statistical analyses in R Studio included Welch’s t-test, chi-square tests, and regression models adjusted for burn severity and demographics. A p-value < 0.05 was considered significant.

**Results:**

Of 3,524 burn encounters, 1,896 adults (34.1% female, 65.9% male) with a mean age of 48.0 years were included. Among these, 297 patients tested positive for cannabis, with use increasing from 2.0% in 2015 to 18.2% in 2024. Cannabis users were predominantly male (77.4% vs 63.8%, p < 0.001) and younger (40.3 vs 49.4, p < 0.001) than non-users, and had a longer hospital LOS (16.8 days vs 11.2 days, p < 0.05). Polysubstance use was identified in 30.3% of cannabis users, with 162 patients (54.5%) also testing positive for amphetamines. When comparing cannabis users (with or without polysubstance use) to non-users, no significant differences were found in burn TBSA, incidence of inhalation injury, ICU and ventilation days, number of operations, graft loss, or inpatient morbidities and mortality. However, cannabis users had higher readmission rates (7.1% vs 0.6%, p < 0.001) and against medical advice (AMA) discharges (12.5% vs 2.2%, p < 0.001). After adjusting for age, gender, race, TBSA, and inhalation injury, cannabis users with polysubstance use had higher odds of AMA discharge (OR 1.065; p < 0.05) compared to non-users, whereas cannabis-only users had lower odds (OR 0.917; p < 0.02). Polysubstance users also had longer LOS (p < 0.05) and ICU days (p < 0.05) compared to cannabis-only users.

**Conclusions:**

We observed a nine-fold increase in cannabis use among the regional adult burn population over the past nine years. While cannabis use was not linked to increased complications or mortality, cannabis-positive patients had significantly higher rates of polysubstance abuse, likely contributing to longer hospital stays and more AMA discharges.

**Applicability of Research to Practice:**

Identifying polysubstance use in burn patients is crucial, as it may impact outcomes, even without direct cannabis-related complications.

**Funding for the Study:**

N/A

